# Invasive *Aspergillus fumigatus *infection after *Plasmodium falciparum *malaria in an immuno-competent host: Case report and review of literature

**DOI:** 10.1186/1475-2875-8-167

**Published:** 2009-07-20

**Authors:** Isabella Eckerle, Damaris Ebinger, Daniel Gotthardt, Ralf Eberhardt, Philipp A Schnabel, Wolfgang Stremmel, Thomas Junghanss, Christoph Eisenbach

**Affiliations:** 1 Section Clinical Tropical Medicine, University Hospital Heidelberg, Im Neuenheimer Feld 324, 69120 Heidelberg, Heidelberg, Germany; 2 Internal Medicine IV, University Hospital, Im Neuenheimer Feld 410, 69120 Heidelberg, Germany; 3 Department of Pulmonology and Critical Care Medicine, Thoraxklinik, Amalienstraße 5, 69126 Heidelberg, Germany; 4 Institute of Pathology, University Heidelberg, Im Neuenheimer Feld 220/221, 69120, Heidelberg, Germany

## Abstract

Invasive fungal infection is rarely reported in association with malaria, even though malaria-associated inhibition of phagocyte function is a well-known condition. Invasive aspergillosis is frequently found in severely immuno-compromised patients but not in healthy individuals. Here, a case of pulmonary invasive aspergillosis in a previously healthy patient with severe *P. falciparum *malaria is presented, who was successfully treated with voriconazol and caspofungin. This is the first survival of malaria-associated invasive aspergillosis.

## Background

There is strong evidence that malaria can lead to altered immune response via modulation of both humoral and cell-mediated immunity. Therefore, *Plasmodium falciparum *malaria with subsequent transient immunosuppression can lead to opportunistic infections in previously immunocompetent patients [1].

Aspergillosis (*Aspergillus *spp.) is a major cause of morbidity and mortality in immuno-suppressed hosts, such as patients with haematological malignancies and transplant recipients. *Aspergillus *spp. is found ubiquitously in the environment worldwide and reaches the alveoli by airborne transmission. In healthy persons, the spores are eliminated by mucociliary clearance and pulmonary macrophages. Although infection in healthy individuals can occur, invasive aspergillosis is extremely uncommon in immuno-competent hosts. There are only four reported cases of invasive aspergillosis complicating falciparum malaria in immuno-competent hosts, all with fatal outcome [2-4].

## Case presentation

A 58-year old Caucasian man returned from an 11-day vacation in the Dominican Republic. He had no previous medical history and no malaria prophylaxis was taken.

Six days after return, he developed a fever of up to 40.5°C and dark-coloured watery diarrhoea with nausea and vomiting. His general practitioner suggested a viral infection and treated him with ibuprofen. The fever remained high and on the 7^th ^day of illness, his general condition deteriorated. On presentation at the hospital, he additionally was complaining of pain upon swallowing.

On admission, he was sleepy, but fully oriented, afebrile and pale. No signs of meningitis were found. The lungs were clear and the heart sounds normal. BP was 150/85 mmHg and the pulse rate 95/min. Blood films showed *P. falciparum *with a parasitaemia of 9.5%. Initial laboratory results were Hb 14.6 g/dl, WBC 8.14/nl, PLT 12/nl, CRP 162.8 mg/l (normal range < 5 mg/l), creatinine 6.09 mg/dl, urea 208 mg/dl, LDH 805 U/l, GOT 69 U/l, GPT 197 U/l. Blood culture, urine culture and serology for hepatitis A, B, C and HIV were negative. Chest x-ray and ultrasound were unremarkable except for mild hepatosplenomegaly. Pharyngitis sicca was diagnosed by the ENT consultant.

Treatment with quinine i.v. was initiated with a loading dose of 7.0 mg/kgBW and continued at a rate of 10 mg/kgBW every eight hours for four hours over 10 days. Parasitaemia decreased to 2.7% within three days and was cleared by day five. He was put on intermittent hemodialysis after developing acute renal failure. Antibiotic coverage with imipenem was started.

On day 5 of hospitalization, respiration deteriorated and the patient was intubated. The chest x-ray revealed patchy infiltrates of the upper left lobe and on bronchoscopy bronchial obstruction with viscous mucus was seen. This was cleared and in the material obtained *Aspergillus fumigatus *was cultured abundantly. The circulating antigens of 0.8 remained below the positive cut-off of 1.0 given by the laboratory. The anti-*Aspergillus *antibody titre remained below the positive cut-off of 1:160 – 1:320. Therapy with voriconazol and caspofungin was started immediately.

**Figure 1:**
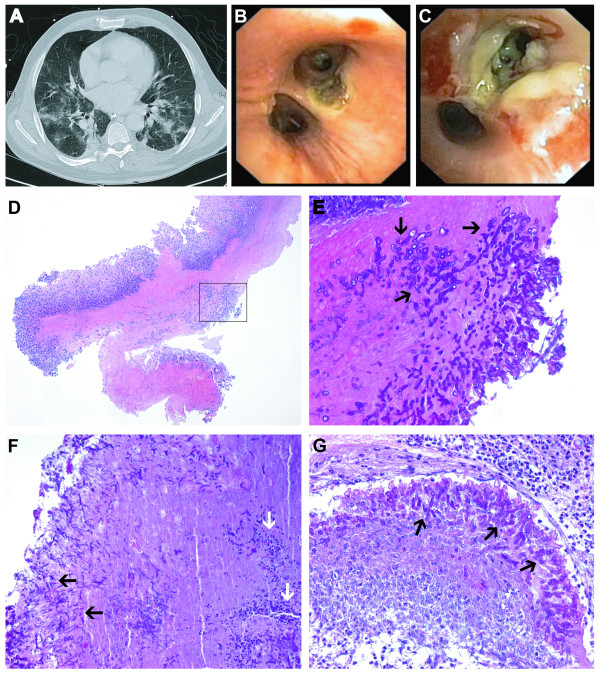
**A: Thoracic CT scan**. Multiple confluent ground glass-infiltrates in both lungs due to *Aspergillus fumigatus*. **B and C: Bronchoscopic findings. B**: Sharply circumscribed lesion of the carina tracheae of the upper left lobe, extending into the upper lobe bronchus. **C**: Extensive necrosis of the bronchial wall extending into the periphery up to segment 2 of the upper right lobe. **D-G: Histological findings of the transbronchial biopsy. D**: Overview of the biopsy specimen, HE stain, primary magnification × 5. The black box indicates the area shown in **E**, primary magnification × 20: PAS staining showing extensive invasive growth of hyphae into the bronchial wall (black arrows). **F**: Mycelia of *Aspergillus fumigatus *with dichotomy branching (black arrows) with inflammatory infiltrates and necrosis (white arrows), PAS stain, primary magnification × 10. **G**: Extensive fungal growth with partially septated hyphae (black arrows), PAS stain, primary magnification × 20.

In the following days respiration improved. On day 7 of artificial ventilation, however, severe haemoptysis suddenly precipitated with cardio-pulmonary arrest. He was successfully resuscitated and pulmonary bleeding spontaneously stopped. A thoracic CT scan showed multiple confluent ground glass-infiltrates of both lungs and enlarged mediastinal and hilar lymph nodes (Figure 1A). Bronchoscopy was performed and showed obstruction of the right main bronchus and lower lobe with coagulated blood. After removal, at the carina tracheae necrotic lesions became apparent and multiple sharply circumscribed ulcerations were found in the bronchi of both upper lobes, suggesting pseudo-membranous necrotizing aspergillosis (Figures 1B and 1C). Pathological examination of the specimen confirmed the diagnosis. PAS-staining showed mycelia with dichotomy branching and partially septated hyphae with extensive invasive growth typical for *Aspergillus *spp. (Figures 1D to 1G). With broad spectrum antibiotic coverage and antifungal therapy with voriconazol and caspofungin, infection parameters decreased, respiration improved and he recovered from an infectious point of view. However, a cranial CT revealed hypoxic brain damage following CPR.

## Conclusion

Aspergillosis, in particular the invasive course of the disease, is only seen in patients with severe immuno-suppression and has a very poor prognosis. In individuals without severe immuno-suppression, invasive fungal infections are extraordinarily rare. Till date invasive aspergillosis during *P. falciparum *malaria has only been described in four cases, none of which survived [2-4]. Two other cases of invasive fungal infections associated with *P. falciparum *malaria had disseminated candidiasis and cryptococcal meningitis [5,6].

*Falciparum *malaria can lead to suppression of both humoral and cell-mediated immunity. Humoral immunity is impaired through reduced production of specific antibodies, while cell-mediated immunity is modulated by inhibition of dendritic cells, which is crucial as they initiate all adaptive and several innate immune responses, and also impairment of T cell and macrophage function plays a role [1,7,8].

Inhibition of key enzymes involved in oxidative burst by the malaria-pigment haemozoin seems to be one of the most important mechanisms contributing to the disruption of macrophage function. Both *in vitro *and *in vivo *studies showed that large proportions of resident macrophages as well as circulating monocytes and leucocytes are loaded with haemozoin and consequently lose their ability to phagocytose and kill ingested germs such as fungi [9]. Especially the latter seems to play a role in invasive fungal infection, because pulmonary macrophages are the major line of defence for *Aspergillus *spp., as they are eliminating spores from the lungs [10]. The central role of phagocytes in the defence of fungal infections is underlined by the following observation: In patients suffering from chronic granulomatous disease, an inherited immunodeficiency due to a NADPH oxidase defect, *Aspergillus *spp. is the most frequent fungal pathogen and pneumonia the major cause of death, because here phagocytes fail to generate reactive antimicrobial oxidants which impairs oxidative burst [11]. The malaria-induced inhibition of macrophages seems to appear late in the acute phase of the disease and persists for various lengths of time after recovery, as haemozoin stays in the affected macrophages for several months [10]. While haemozoin induces functional inhibition of macrophages, the availability of macrophages at the site of infection (in the case presented the lung) is questionable, as during malaria infection macrophages in the blood are likely concentrated where parasitized red blood cells are concentrated. These organs include spleen, liver and bone marrow, but not the lung.

In the case presented, as in the four other published cases, inhibition of macrophages leading to aspergillosis was most likely malaria-associated as no other cause was found. Other mechanisms such as inhibition of humoral and antigen-specific T-cells response are unlikely to be a strong factor for malaria-induced immunosuppression in the acute phase of the disease in a patient without prior malaria infection or semi-immunity. Prolonged and profound neutropenia, the single most important and common risk factor for invasive aspergillosis, was neither present in this case nor in the patients published.

In contrast to the previously described fatal cases, no other malaria-related complication such as cerebral malaria or ARDS was seen in this case, but in two of the other published cases. The patient responded quickly to malaria treatment with rapid clearance of parasites. Invasive aspergillosis became clinically apparent only after malaria parasites had been eradicated and after the patient had started to improve already which is not surprising, as it is known that malaria-induced immunosuppression occurs late in the acute phase of the disease. Whether the initial presentation of pain upon swallowing was already related to the beginning of the *Aspergillus *infection is not clear. The *Aspergillus *antigen detection with a magnitude below 1.0 was interpreted as negative despite extensive *Aspergillus *growth in the culture. However, in many settings now a positive cut-off of 0.5 is used, therefore the maximal antigen magnitude of 0.8, which was measured after the positive culture result, would meet the criteria for a positive result. There are also several studies which showed that galactomannan ELISA is not as sensitive in invasive aspergillosis as anticipated [12]. The negative anti-*Aspergillus *antibody titre could be an additional marker for severely impaired immune response to *Aspergillus *due to malaria-induced immunosuppression.

Voriconazole is a triazol antifungal that is considered first-line-treatment for invasive aspergillosis [13]. Because of the progressive and life-threatening course of the disease, caspofungin, which belongs to the echinocandins, a new class of anti-fungals, was administered additionally. Early recognition and immediate treatment with two potent anti-fungals may have been the key to successful treatment of invasive aspergillosis and the first described survival of a patient.

As malaria mostly occurs in low-resource countries with limited access to diagnostic tools, underreporting of fungal disease complicating malaria may be a problem. With only four cases reported so far, the incidence seems to be very low, however. For practical purposes invasive fungal disease should be considered in malaria patients deteriorating after successful anti-malarial treatment. This case shows that early recognition and immediate treatment in principle can avert a fatal outcome.

## Consent

Written informed consent was obtained from the patient for publication of this case report and any accompanying images. A copy of the written consent is available for review by the Editor-in-Chief of this journal.

## Competing interests

The authors declare that they have no competing interests.

## Authors' contributions

IE collected all information and wrote the first version of the manuscript. IE and DG reviewed the literature. RE and PS supplied macroscopic and microscopic photographic material. All authors gave their input into the final version of the manuscript. DE, CE and TJ were the physicians responsible for the patient. All authors read and approved the final manuscript.
